# Processing of Spent Titanium Chlorinator Melt for Scandium Recovery

**DOI:** 10.3390/ma19173652

**Published:** 2026-08-27

**Authors:** Almagul Ultarakova, Azamat Yessengaziyev, Nina Lokhova, Bauyrzhan Orynbayev, Azamat Toishybek, Arailym Mukangaliyeva, Kaisar Kassymzhanov

**Affiliations:** 1 The Institute of Metallurgy and Ore Beneficiation JSC, Satbayev University, Almaty 050013, Kazakhstan; a.ultarakova@satbayev.university (A.U.); n.lokhova@satbayev.university (N.L.); azxoff@gmail.com (A.T.); a.mukangaliyeva@satbayev.university (A.M.); k.kassymzhanov@satbayev.university (K.K.); 2 Laboratory of Metallurgical Processing of Materials, K. Zhubanov Aktobe Regional University, Aktobe 030000, Kazakhstan; b.orynbayev@zhubanov.edu.kz

**Keywords:** scandium, spent titanium chlorinator melt, hydrochloric acid leaching, cation-exchange sorption, Lewatit SP112H, scandium oxide

## Abstract

Spent titanium chlorinator melt is a potential technogenic source of scandium, but its recovery is complicated by high contents of iron, aluminium and alkali and alkaline-earth chlorides. This study assessed a sequence of hydrochloric acid leaching, Fe(III) reduction with ascorbic acid, sorption on the strong-acid cation exchanger Lewatit MonoPlus SP112H, desorption with acidified ammonium sulfate solution, oxalate precipitation, calcination and an additional column purification. In a scaled-up test, leaching of 4 kg of the spent melt with 5% HCl produced 25.5 L of filtrate containing 475.30 mg Sc. Two-stage sorption recovered 90.9% of the scandium, and the overall desorption degree reached 94.8%. The kinetic data were better described by the non-linear pseudo-second-order model (*R*^2^ = 0.890–0.938). Precipitation and calcination gave 0.60 g of an intermediate oxide product with 65.0 wt% Sc, and the additional purification 0.45 g of a final oxide product in which X-ray diffraction identified cubic Sc_2_O_3_ as the predominant crystalline phase; the elemental analysis gave a purity close to 98 wt%. Recovery into the final oxide reached 61.92% of the scandium in the filtrate. Iron co-sorption and scandium losses during the additional purification remained the principal limitations of the process.

## 1. Introduction

Scandium is regarded as a critical raw material because of its applications in Al–Sc alloys and solid oxide fuel cells, while its primary supply remains limited [1,2,3,4]. Since scandium rarely forms economically mineable minerals, it is recovered mainly as a by-product from complex ores, residues and previously processed wastes [5]. Among technogenic sources, titanium–magnesium production wastes and bauxite-processing residues are of particular interest [6,7]. The scandium content of titanium-production wastes may reach 100–350 g/t [8], which makes these materials prospective secondary feedstocks for scandium recovery.

Titanium–magnesium plants treat ilmenite concentrate by ore-thermal smelting followed by chlorination. Up to 97% of the scandium reports to the titanium slag during smelting. During chlorination of the slag in an MgCl_2_–KCl–NaCl melt, about 83.8% of the scandium concentrates in the solid spent chlorinator phase, and only about 5.7% leaves with the vapour–gas stream. UKTMP JSC generates 30–35 thousand t of solid chloride titanium-production wastes annually [8]. Spent titanium chlorinator melt (STCM) remains a realistic scandium source for Kazakhstan [9].

Scandium is leached from the chloride residue with hydrochloric acid and passes into solution in cationic form [10]. The resulting solutions are lean in scandium and contain a manifold excess of iron, titanium, zirconium and aluminium [11,12]. Pregnant leach solutions combine a high salt background, an acidity below pH 2 and a low concentration of the target metal. The similarity of scandium and iron in charge, ionic radius and hardness defines the main difficulty of selective separation. Co-extraction and co-precipitation of iron, and not the choice of reagent, limit the purity of the scandium product [13].

Precipitation, solvent extraction and ion exchange are used to concentrate scandium [14]. Extraction with organophosphorus reagents provides high recoveries [14,15]. Di-(2-ethylhexyl)phosphoric acid, Cyanex 923 and tributyl phosphate recover scandium at a purity of 95–96% in 6–9 extraction and scrubbing stages [11,15]. The recovery of valuable metals from complex metallurgical wastes can also be intensified by preliminary modification of their phase composition [16]. Roasting of ilmenite slag with Na_2_CO_3_ at about 950 °C, leaching with 30% hydrochloric acid at 80 °C and extraction with 30% D2EHPA recover 94% of the scandium from the leachate at 2.2% iron co-extraction, while precipitation with oxalic acid and calcination at 800 °C yields Sc_2_O_3_ of 99% purity [17]. Stripping frequently requires concentrated alkalis or fluorides and produces poorly filterable precipitates [14]. Extractant losses, emulsification and iron co-extraction reduce the economics of the flowsheet [13,18].

Ion exchange has been considered a promising approach for scandium recovery from acidic leaching solutions. Strong-acid cation-exchange, chelating and solvent-impregnated resins have been investigated for this purpose [19]. Solvent-impregnated resins take up scandium selectively from dilute solutions that carry high impurity loads, but the extractant is gradually lost to the aqueous phase and elution of the metal remains difficult. On TVEX resins scandium is recovered from hydrochloric acid solutions stronger than 4 M, which confines their use to strongly acidic media [20]. In acidic media strong-acid cation exchangers display the selectivity series REE ≈ Th > Fe ≈ Al and retain heavy lanthanides and scandium more weakly [21]. On sulfonic cation exchangers, uptake proceeds by cation exchange, in which the trivalent scandium ion displaces the protons of the fixed sulfonic groups. The exchange stoichiometry governs the competition for sorption sites: at high concentrations of hydrogen ions and trivalent iron the equilibrium shifts against scandium fixation, which limits selectivity in strongly acidic chloride solutions [22]. Reduction of iron to the divalent state before sorption is the practical way of lowering this competition. In [19], a feed solution containing 12.7 mg/L Sc and 272 mg/L Fe was treated with iron powder to decrease the Fe(III) content.

For chloride solutions of titanium production, scandium sorption on commercial ion exchangers at room temperature has been reported. No appreciable iron sorption occurred on the phosphorus-bearing resins VP OC 1026 and TP 260, and these resins separated scandium from iron, leaving the latter in solution [23]. Peng et al. demonstrated stagewise purification of a scandium chloride solution from Fe, Zr, Ti and Al through control of acidity [24]. Yessimkanova et al. examined the kinetics of scandium sorption on the resin Purolite MTS9580 [25]. Napol’skikh et al. recovered scandium from sulfuric acid solutions of bauxite processing on the resin Puromet MTS9580 [26].

In a previous study the strong-acid cation exchangers KU-2-8, Lewatit SP112H, Purosorb SAC140H and Purolite C-150H were compared in acidic chloride solutions and the sorption equilibrium of scandium was examined. Among the materials studied, Lewatit SP112H showed the highest affinity for scandium, which provided the basis for its selection in the present work [8]. Results obtained in controlled solutions, however, do not allow the behaviour of the resin in an STCM leach filtrate to be assessed directly, since the iron concentration there exceeds the scandium content by several orders of magnitude. The distribution of scandium between the successive stages of leaching, sorption, desorption, precipitation and calcination had not been traced either.

The cost of scandium production is strongly influenced by the feedstock decomposition stage. Therefore, by-product recovery of scandium at an operating titanium plant may be more economically attractive because scandium is already concentrated in the process residues. The aim of this work was to experimentally evaluate an integrated scandium recovery sequence from real spent titanium chlorinator melt. To achieve this aim, the following objectives were defined:

(i). to determine scandium recovery and the co-sorption of Fe and Al;
(ii). to calculate the separation factors and resin loading;
(iii). to establish the stagewise distribution and losses of scandium from the combined leach filtrate to the final oxide product.

The novelty of the work lies in the stagewise evaluation of the proposed recovery sequence using a real STCM-derived process solution, including scandium distribution and losses during leaching, sorption, desorption, oxalate precipitation, calcination, and additional purification.

## 2. Materials and Methods

### 2.1. Materials, Equipment and Analytical Methods

The object of the study was spent titanium chlorinator melt from titanium–magnesium production. One averaged batch of material was used for the laboratory and the scaled-up tests. Before the experiments, the STCM was mixed and ground in a laboratory ball mill with ceramic grinding media.

The elemental composition of the material is given in Table 1. The contents of Sc, Fe and Al were 0.015, 9.02 and 1.01 wt%, respectively.

Sorption experiments were carried out batchwise in a shaker incubator using the commercial strong-acid macroporous monodisperse cation exchanger Lewatit MonoPlus SP112H in the H^+^ form (Lanxess AG, Cologne, Germany). According to the manufacturer’s technical information, the resin is based on a sulfonated styrene–divinylbenzene matrix. Its total exchange capacity is at least 1.6 eq/L of wet resin, the mean bead size is 0.67 ± 0.05 mm, and the water retention is 56–60% [27].

The experiments employed a BML-2 laboratory ball mill (DAIHAN Scientific, Wonju-si, Republic of Korea), an IKA KS 3000 i control shaker incubator (IKA-Werke, Staufen, Germany), a SNOL 58/350 LFN drying oven (SNOL, Utena, Lithuania) and an LF-7/13-G1 muffle furnace (LOIP, St. Petersburg, Russia).

**Figure 1:**
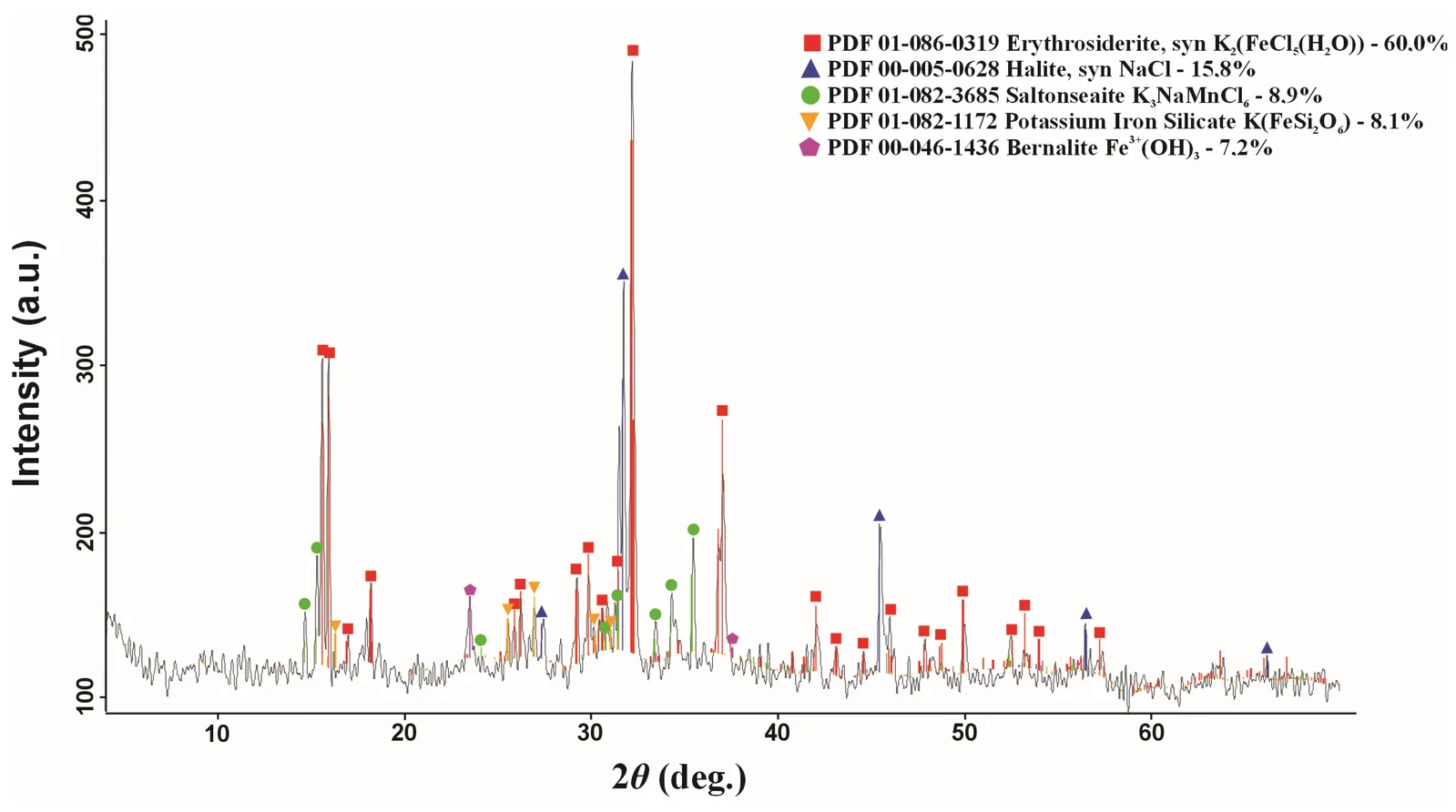
X-ray diffraction pattern of the STCM with semi-quantitative phase composition.

The concentrations of Sc, Fe and Al in solutions were determined by inductively coupled plasma optical emission spectrometry on an Optima 8300 DV spectrometer (PerkinElmer, Waltham, MA, USA). The phase composition of the initial STCM was determined by powder X-ray diffraction on a D8 ADVANCE diffractometer (Bruker, Karlsruhe, Germany) using Cu Kα radiation. The diffraction pattern of the initial STCM is shown in Figure 1. The content of components was also determined by X-ray fluorescence spectrometry on an Axios spectrometer (PANalytical, Almelo, The Netherlands).

Erythrosiderite K_2_FeCl_5_·H_2_O, halite NaCl, saltonseaite K_3_NaMnCl_6_, the iron-bearing silicate KFeSi_2_O_6_ and bernalite Fe(OH)_3_ were identified in the material. Chloride compounds of potassium, sodium, iron and manganese prevail, with silicate and iron hydroxide phases also present.

### 2.2. Laboratory Leaching and Sorption Tests

The effect of hydrochloric acid concentration was studied on 80 g STCM samples. Leaching was performed at a solid-to-liquid ratio of 1:6, a temperature of 90 °C, a duration of 60 min and a stirring speed of 300 rpm. The HCl concentration was varied from 2 to 15%. After the process the pulp was filtered under vacuum and the filtrates were analysed for Sc, Fe and Al.

To evaluate the subsequent sorption, 100 mL of each filtrate was contacted with 3 mL of swollen Lewatit SP112H at room temperature and 200 rpm for 30 min. Since a change in HCl concentration simultaneously affected the dissolution of the STCM components, the residual acidity and the filtrate composition, the results describe the combined leaching–sorption sequence.

The effect of resin dosage was studied on the filtrate obtained by leaching STCM with 5% HCl. The tests used 3 mL of swollen Lewatit SP112H at resin-to-solution volume ratios of 1:100 to 1:500. Sorption was carried out at room temperature and 200 rpm for 30 min.

For the dilution study the pregnant solution was obtained by leaching 40 g of STCM with 5% HCl at a solid-to-liquid ratio of 1:10, a temperature of 80 °C, a duration of 90 min and a stirring speed of 300 rpm. The filtrate was diluted with distilled water to the required scandium concentrations. Each test received 1 mL of swollen Lewatit SP112H, and sorption proceeded at room temperature for 6 h.

Sorption kinetics were studied at 20, 40, 60 and 80 °C over 15–540 min. The kinetic curves were fitted with the non-linear forms of the pseudo-first-order and pseudo-second-order models. The contribution of intraparticle mass transfer was evaluated with the Weber–Morris model. Fit quality was compared using the coefficient of determination and the root-mean-square error.

All experiments were run in three independent replicates. The results are reported as the arithmetic mean ± standard deviation.

### 2.3. Scaled-Up Test and Preparation of the Products

The scaled-up test was performed on 4.0 kg of STCM. Leaching was carried out with 5% HCl at a solid-to-liquid ratio of 1:6, a temperature of 80–90 °C, for a duration of 60 min and with a stirring speed of 300 rpm. Ascorbic acid was added to the pulp in portions to reduce part of the Fe^3+^ to Fe^2+^; the total reagent consumption was 108 g. The dose was set empirically from the colour change of the pulp on reduction of Fe(III) to Fe(II). This amount corresponds to 0.61 mol of ascorbic acid, equivalent as a two-electron reductant to the reduction of about 68 g of iron, close to one third of the 183.75 g of iron in the combined filtrate. No independent measurement of the Fe(III)/Fe(II) ratio or redox potential was performed; therefore, the extent of Fe(III) reduction was not directly quantified, and the calculated value represents the theoretical reducing capacity of the added reagent rather than an experimentally verified degree of reduction.

After leaching, the pulp was filtered under vacuum. The cake was washed with 1.5 L of hot distilled water, and the wash solution was combined with the main filtrate. The total volume of the combined filtrate was 25.5 L.

Scandium was recovered in two successive sorption stages on the strong-acid cation exchanger Lewatit MonoPlus SP112H. The first stage used 245 mL of swollen resin, corresponding to 185.22 g of dry resin, and the second stage used 80 mL, or 60.5 g on a dry basis. Both sorption stages were run batchwise at 80 °C for 6 h at a stirring speed of 200 rpm.

The loaded resin was washed with 1–2% HCl and then with distilled water and subjected to desorption with an ammonium sulfate solution of 200 g/L acidified with sulfuric acid to pH 1.5–2.0. Desorption was carried out at a resin-to-eluent volume ratio of 1:2, a temperature of 45 °C and a duration of 6 h.

Scandium was precipitated from the primary eluate with oxalic acid dihydrate at a 1.5–2-fold excess over the stoichiometric amount. The oxalate precipitate was filtered off, washed, dried at 100 °C for 120 min and calcined to give an intermediate oxide product. For additional purification the oxide was dissolved in 15% HCl, the solution was diluted to an HCl concentration of 5%, and scandium was recovered again on a column packed with 20 mL of Lewatit MonoPlus SP112H. After an acid wash of the resin, scandium was desorbed with an ammonium sulfate solution of 200 g/L acidified with sulfuric acid to pH 1.5–2.0. Scandium was reprecipitated from the purified eluate with oxalic acid dihydrate, and the precipitate was dried and calcined at 800 °C to give the final Sc_2_O_3_.

The raffinate from the two sorption stages was neutralised with lime milk to pH 4–5. After the precipitate had been separated, MgCl_2_ was added to the purified solution to a KCl/MgCl_2_ mass ratio of 1.2, and the solution was evaporated at 80 °C. The mixed potassium–sodium–magnesium chloride product that crystallised was filtered off and dried at 100 °C.

### 2.4. Calculations and Statistical Treatment

The recovery of an element onto the sorbent was calculated as:

(1) $$R=\frac{C_{0}-C_{e}}{C_{0}}\times 100\%$$

where *C*_0_ and *C*_e_ are the initial and equilibrium concentrations of the element, mg/L.

The sorption capacity of the resin was determined as:

(2) $$q_{e}=\frac{\left(C_{0}-C_{e}\right)V}{m}$$

where *V* is the solution volume, L, and *m* is the dry resin mass, g. The capacity *q*_t_ at time *t* was calculated from Equation (2) with the equilibrium concentration *C*_e_ replaced by the current concentration *C*_t_.

The distribution coefficient was calculated as:

(3) $$K_{d}=\frac{C_{0}-C_{e}}{C_{e}}\cdot \frac{V}{m}$$

and the separation factor of scandium over an impurity element Me as:

(4) $$\beta_{Sc/Me}=\frac{K_{d,Sc}}{K_{d,Me}}=\frac{R_{Sc}\left(100-R_{Me}\right)}{R_{Me}\left(100-R_{Sc}\right)}$$

since at equal solution volume and sorbent mass, the ratio of *V* to *m* cancels and the separation factor is expressed directly through the recoveries of the elements. The *K*_d_ values are given in mL/g.

The kinetic curves were fitted with the non-linear forms of the pseudo-first-order and pseudo-second-order models [28,29]:

(5) $$q_{t}=q_{e}\left(1-e^{-k_{1}t}\right),$$

(6) $$q_{t}=\frac{k_{2}q_{e}^{2}t}{1+k_{2}q_{e}t},$$

where *q*_t_ and *q*_e_ are the sorption capacities at time *t* and at equilibrium, mg/g; *k*_1_ is the pseudo-first-order rate constant, min^−1^; and *k*_2_ is the pseudo-second-order rate constant, g·mg^−1^·min^−1^. The initial sorption rate was defined as:

(7) $$h=k_{2}q_{e}^{2}.$$

The contribution of intraparticle mass transfer was evaluated with the Weber–Morris model [30]:

(8) $$q_{t}=k_{id}t^{1/2}+C,$$

where *k*_id_ is the intraparticle diffusion coefficient, mg·g^−1^·min^−1/2^, and *C* is the intercept, which characterises the deviation of the plot from the origin. The apparent activation energy was estimated from the temperature dependence of the initial sorption rate in Arrhenius coordinates:

(9) $$lnh=lnA-\frac{E_{a}}{RT},$$

where *A* is the pre-exponential factor; *E*_a_ is the apparent activation energy, J/mol; *R* = 8.314 J·mol^−1^·K^−1^; and *T* is the temperature, K.

The parameters of the kinetic models were determined separately for each temperature by non-linear regression. Agreement between calculated and experimental values was assessed from the coefficient of determination *R*^2^.

## 3. Results and Discussion

### 3.1. Effect of Hydrochloric Acid Concentration

The hydrochloric acid concentration affects both the dissolution of the STCM components and the subsequent distribution of the metals between solution and resin. The results of this series therefore describe the combined leaching–sorption sequence rather than the isolated effect of acidity on ion exchange.

**Figure 2:**
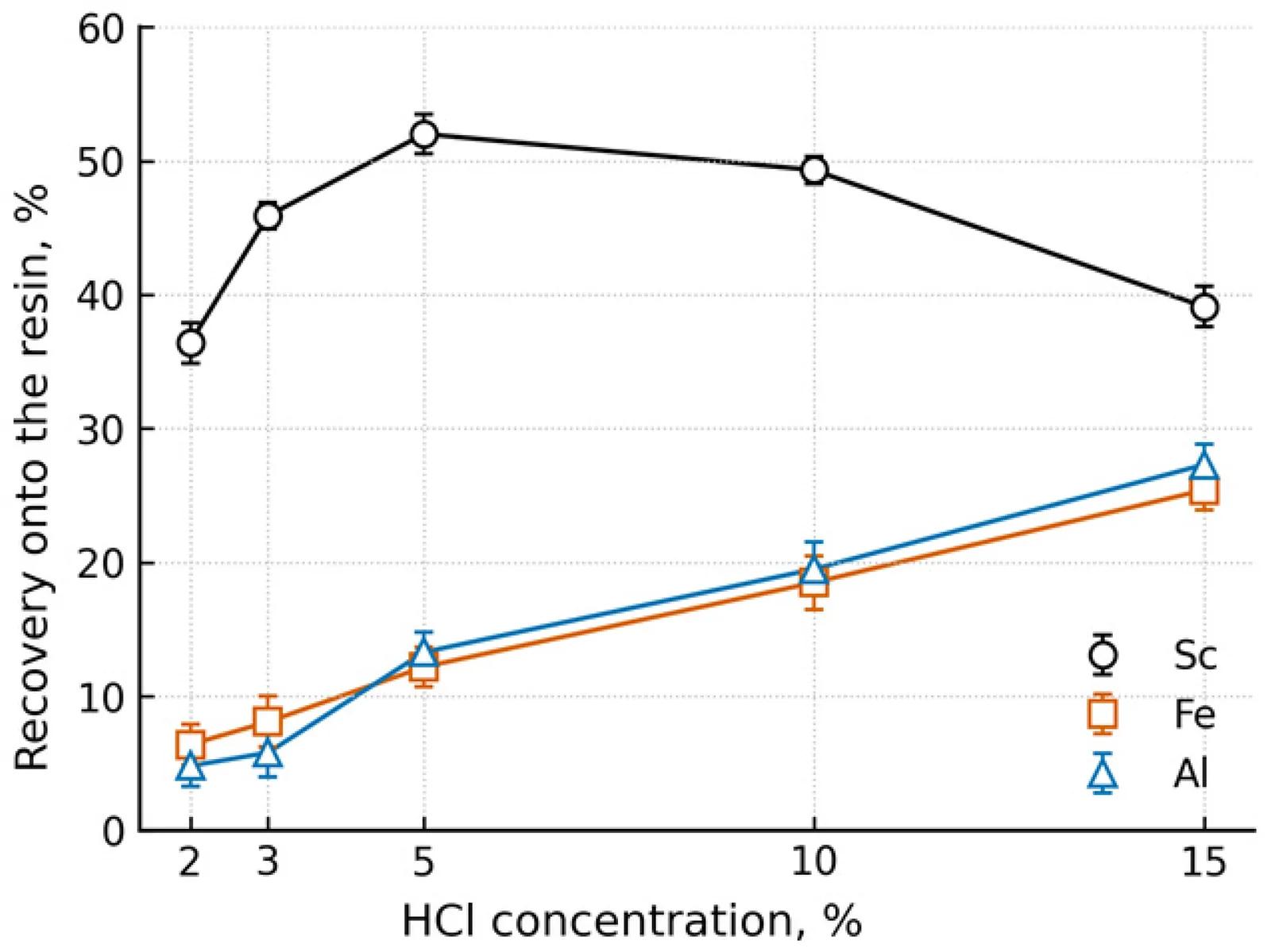
Effect of the HCl concentration used in leaching on the recovery of Sc, Fe and Al on Lewatit SP112H.

As the HCl concentration rose from 2 to 5%, scandium recovery on Lewatit SP112H increased from 36.4 to 52.0% (Figure 2). A further increase in acid concentration did not improve sorption: at 10% HCl the recovery of scandium was about 49%, and at 15% it fell to 39.1%. The maximum scandium loading of the resin (1.107 mg/g) was reached for the filtrate obtained at 10% HCl.

The observed dependence arises from several factors acting together. Below 5% HCl, the transfer of scandium from the STCM into solution is limited, which lowers its content in the filtrate. At higher acidity the H^+^ concentration rises and the chloride complexation of the metals changes, which affects the distribution of Sc, Fe and Al between the solution and the sulfonic groups of the resin. Co-sorption of impurities increases at the same time: at 15% HCl about 25% of the iron and 27% of the aluminium passed onto the resin.

The initial scandium concentration in the filtrate also passed through a maximum in the 5–10% HCl range. At 5% HCl it was 48.34 mg/L and at 3% HCl 42.96 mg/L, whereas at 2 and 15% HCl it was 33.00 and 34.88 mg/L, respectively. The lower sorption capacity at the extreme acidities is therefore caused not only by a change in the affinity of the resin but also by a smaller amount of scandium available for sorption.

Separation factors of scandium over iron and aluminium were calculated to assess selectivity. The results are given in Table 2.

The highest selectivity was reached at 3% HCl: β_Sc/Fe_ = 9.6 and β_Sc/Al_ = 13.8. At 5% HCl the separation factors decreased to 7.8 and 7.1, while scandium recovery was about six percentage points higher and the resin loading rose from 0.508 to 0.870 mg/g. At 10% HCl, where the loading was highest, recovery decreased and the separation factors fell to 4.3 and 4.0. At 15% HCl β_Sc/Fe_ and β_Sc/Al_ fell to 1.9 and 1.7, which indicates a loss of selectivity caused by stronger co-recovery of iron and aluminium. Recovery and selectivity thus respond to acidity in opposite directions, and 5% HCl was selected for the scaled-up experiment as a compromise between scandium recovery, resin loading, acid consumption and the mass of co-sorbed iron and aluminium.

The value of 5% HCl was taken from this single-factor series and is not a formally optimised value. The acid concentration was varied in discrete steps of 2, 3, 5, 10 and 15% without statistical experimental design or multivariable optimisation, and intermediate concentrations were not examined. The 3% regime merits separate evaluation where separation from impurities has priority over throughput.

Because changing the HCl concentration simultaneously changes acidity, chloride concentration and the composition of the leach solution, the present experiments do not separate the individual effects of pH and competing anions. Independent variation in pH and anion composition was outside the scope of the present study and should be addressed in a dedicated mechanistic investigation.

### 3.2. Effect of Resin Dosage and Dilution of the Pregnant Solution

**Figure 3:**
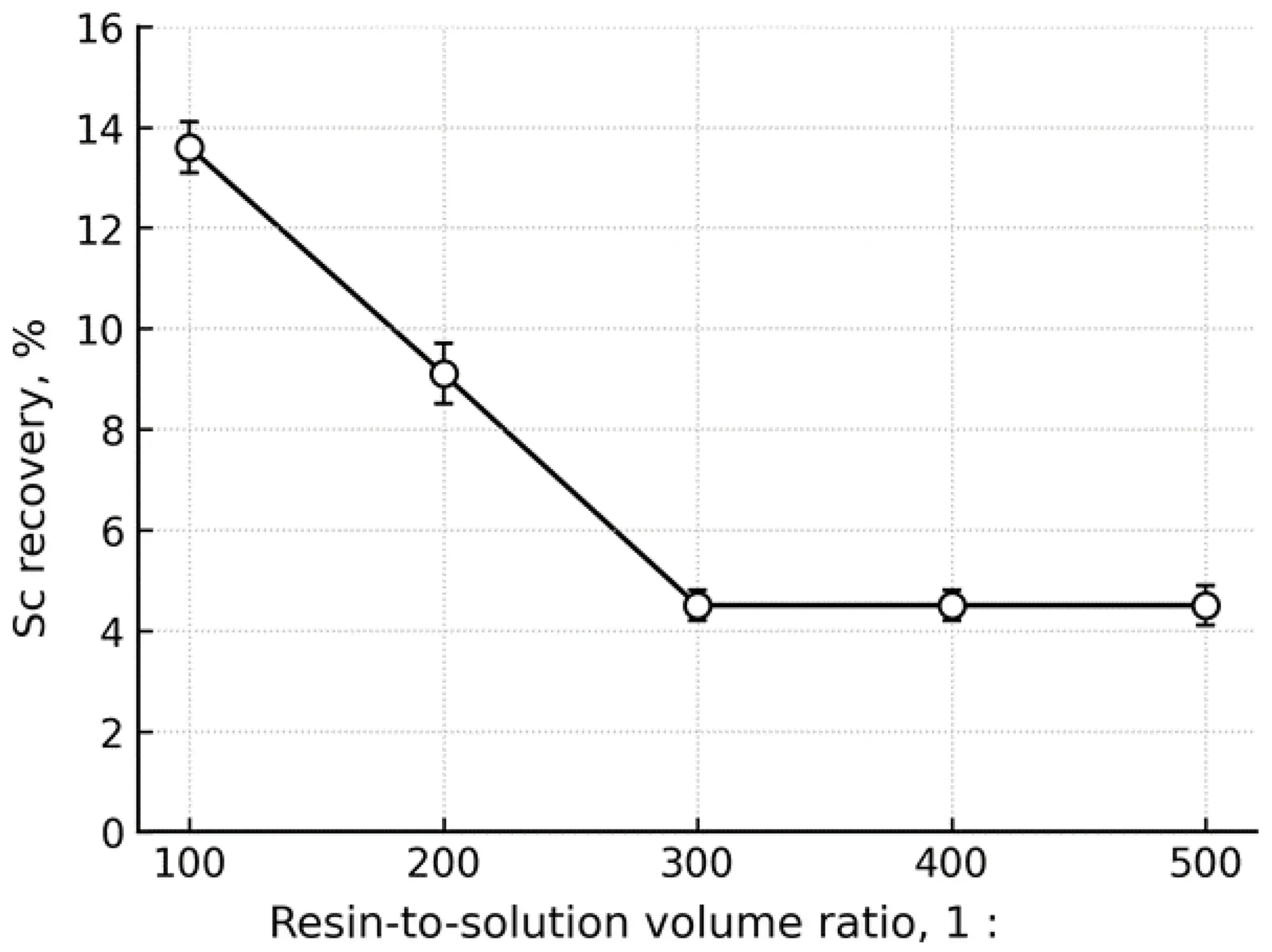
Effect of the phase ratio (resin volume to solution volume) on scandium recovery (mean of three tests, error bars ± SD).

The resin dosage determines the fraction of available ion-exchange sites relative to the amount of dissolved metals. When the volume ratio of swollen resin to solution was raised from 1:500 to 1:100, scandium recovery increased from 4.5 to 13.6% (Figure 3). The largest change occurred on going from 1:300 to 1:100, whereas at ratios of 1:300–1:500 the recovery remained near 4.5%.

The low recovery compared with Section 3.1 is due mainly to the smaller resin dosage. In the acidity series, 3 mL of resin was contacted with 100 mL of solution, which corresponds to 30 mL of resin per L. In the phase-ratio tests, the dosage varied from 2 to 10 mL/L. A smaller number of available sulfonic groups relative to the amounts of scandium and competing cations naturally reduced the recovered fraction of scandium.

Direct quantitative comparison of the two series is limited by differences in the composition of the initial solutions. Assessment of dosage efficiency requires recovery and specific resin loading to be considered together. An increased resin dose may raise recovery while lowering *q*_Sc_ because the exchange capacity is used incompletely. The choice of phase ratio must therefore rest not only on maximum recovery but also on resin consumption and the subsequent scandium concentration in the eluate.

**Figure 4:**
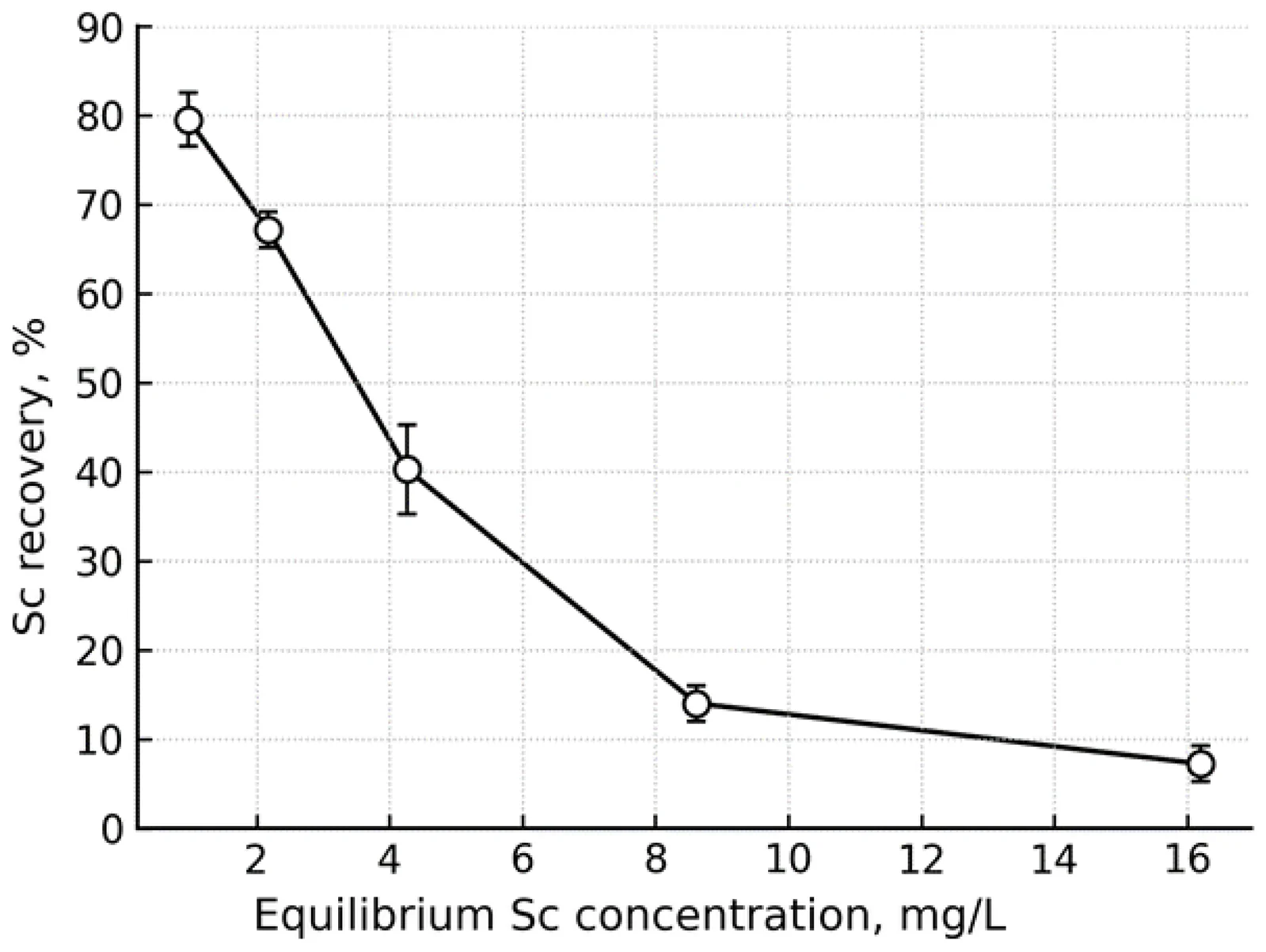
Scandium recovery as a function of the equilibrium Sc concentration.

A separate series examined sorption from solutions obtained by successive dilution of a single filtrate. As the scandium concentration fell from 16.2 to 0.98 mg/L, its recovery rose from 7.22 to 79.59% (Figure 4). The specific resin loading was 0.155 mg/g for the most concentrated solution. The higher recovery on dilution reflects the larger share of scandium per fixed number of sorption sites and does not mean an increase in sorbent capacity. The ability of the resin to scavenge scandium efficiently from dilute solutions matters for the polishing of the barren solutions after the main sorption stage.

The higher recovery on dilution does not indicate an increased equilibrium affinity of Lewatit SP112H for scandium. At a constant resin dosage, the total mass of scandium in solution decreased, so the available exchange sites sufficed to take up a larger fraction of the metal. The concentrations of Fe, Al, HCl and background chlorides fell together with that of scandium, which further weakened the competition for the sulfonic groups. The percentage recovery consequently rose while the absolute resin loading decreased.

This series is therefore not a classical sorption isotherm, because dilution changed the acidity, ionic strength and concentrations of all solution components. The data describe the behaviour of the resin as the total salinity of the pregnant solution decreases.

The practical value of the results lies in the scavenging of scandium from the depleted raffinate. Deliberate dilution of the initial filtrate increases the volume of solution to be treated and the water consumption and is therefore not attractive technologically. Adding a further portion of resin after the main sorption stage, when the concentrations of scandium and of the competing components are already low, is better justified. This approach was used in the scaled-up test.

### 3.3. Sorption Kinetics and Selection of the Contact Time

**Figure 5:**
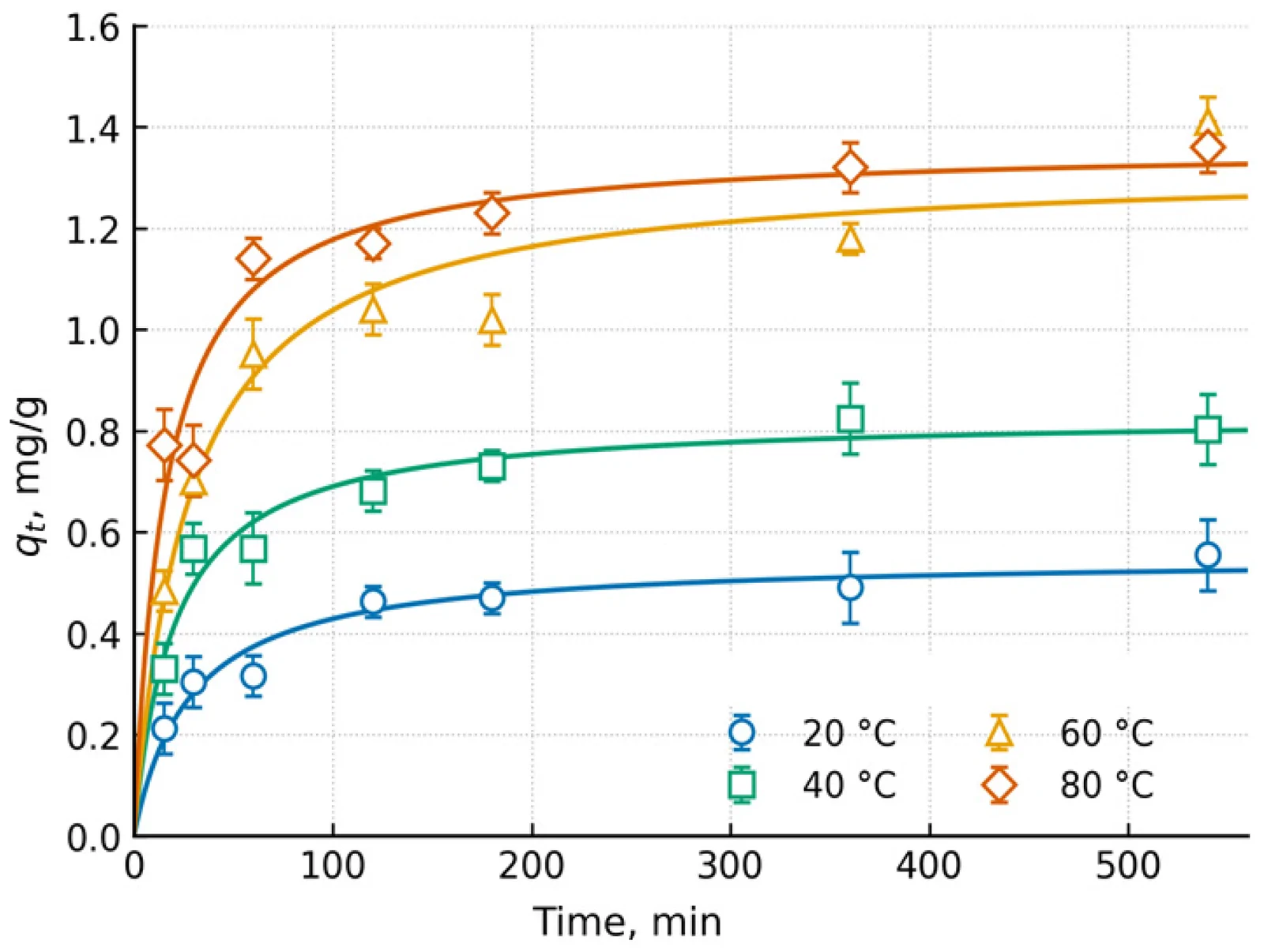
Change in the sorption capacity *q*_t_ with time.

The kinetics of scandium sorption on Lewatit SP112H were studied at 20, 40, 60 and 80 °C over contact times of 15–540 min (Figure 5). The sharpest increase in sorption capacity occurs during the first 120 min, after which scandium accumulates more slowly. By 540 min, the capacity reaches 0.555, 0.803, 1.410 and 1.360 mg/g at 20, 40, 60 and 80 °C, respectively. Individual non-monotonic deviations of the experimental points do not exceed the standard deviation of the replicate tests and do not disturb the general trend of increasing loading with time.

A higher temperature increases the resin loading attained over the period studied. This result reflects faster mass transfer on heating but does not establish the thermodynamic character of sorption: at 20 °C the system may not have reached equilibrium within 540 min. Calculation of the enthalpy of the process requires equilibrium distribution coefficients measured at each temperature.

The experimental *q*_t_(*t*) curves were fitted with the non-linear forms of the pseudo-first-order and pseudo-second-order models, with the parameters calculated separately for each temperature and without prior linearisation of the equations (Table 3). The pseudo-second-order model describes the data more accurately: the coefficient of determination is 0.890–0.938 against 0.785–0.853 for the pseudo-first-order model, the root-mean-square deviation is lower by a factor of 1.4–1.5, and the Akaike information criterion is smaller by 4.7–6.1 units at an equal number of parameters. The calculated equilibrium capacity rises from 0.551 mg/g at 20 °C to 1.364 mg/g at 80 °C, and the initial sorption rate *h* increases from 0.0193 to 0.0858 mg·g^−1^·min^−1^. The constant *k*_2_ varies non-monotonically and cannot therefore be used for a direct estimate of the activation energy from the Arrhenius equation.

The better fit of the pseudo-second-order model characterises its suitability for the mathematical description of the kinetic curves but does not establish the sorption mechanism. The model formally describes processes governed jointly by ion exchange, external mass transfer and diffusion inside the beads, so a conclusion about the rate-limiting step requires diffusion models.

The contribution of intraparticle transport was evaluated with the Weber–Morris model. The plots of *q*_t_ against *t*^1/2^ do not pass through the origin: the intercept *C* is 0.209–0.736 mg/g and increases with temperature, while the intraparticle diffusion coefficient *k*_d_ varies within 0.0161–0.0400 mg·g^−1^·min^−1/2^ at *R*^2^ = 0.773–0.880. Intraparticle diffusion takes part in the transport of scandium but is not the only rate-controlling step. The non-zero intercept is consistent with an additional contribution of external mass transfer through the boundary film at the bead surface.

The apparent activation energy was estimated from the temperature dependence of the initial sorption rate *h* in Arrhenius coordinates; linear fitting over four temperatures gave *E*_a_ = 20.0 kJ/mol at *R*^2^ = 0.956. The quantity *h* is not an elementary rate constant, so the value obtained should be treated as an effective measure of the temperature sensitivity of the initial sorption stage rather than as the activation energy of a single step. A value below 40 kJ/mol is not inconsistent with the predominance of diffusion control indicated by the non-zero intercept of the Weber–Morris model.

The contact time was selected from the loading attained. By 360 min, the experimental capacities amount to 89.1, 99.3, 89.1 and 96.8% of the equilibrium capacities calculated from the pseudo-second-order model at 20, 40, 60 and 80 °C. At 60 °C the capacity continues to grow appreciably after 360 min, from 1.18 to 1.41 mg/g by 540 min, that is by 19.5% at a standard deviation of 0.05 mg/g. A duration of 6 h was adopted as a practical compromise between the loading attained and the length of the operation, and not as a strictly established time to equilibrium. Equilibrium was not experimentally confirmed at all temperatures within the investigated time range. Accordingly, the fitted qe values should be regarded as model-estimated capacities rather than independently verified equilibrium capacities.

### 3.4. Scaled-Up Test, Scandium Mass Balance and Products

Leaching and washing of the cake produced 25.5 L of a combined filtrate containing 475.30 mg Sc, 183.75 g Fe and 0.98 g Al. The mass balance of the subsequent operations was calculated relative to the mass of scandium in the combined filtrate.

The first sorption stage transferred 379.29 mg Sc to the resin, corresponding to a recovery of 79.8%. The scandium capacity of the resin was 2.05 mg/g. At the same time, 9.573 g Fe and 31 mg Al were sorbed, corresponding to recoveries of 5.21 and 3.17%. The iron and aluminium capacities of the resin were 51.68 and 0.17 mg/g, respectively. The low iron uptake is consistent with the prior reduction of Fe(III) to Fe(II), which is held far less strongly by the sulfonic groups.

Despite the comparatively low iron recovery, the absolute iron content of the loaded resin greatly exceeded the scandium content because of the high initial iron concentration in solution. The Fe:Sc mass ratio decreased from about 386:1 in the initial filtrate to 25:1 in the loaded resin. The first sorption stage thus provided an approximately fifteen-fold enrichment of scandium relative to iron. The sorption concentrate nevertheless remained predominantly iron-bearing, which points to the limited selectivity of the strong-acid cation exchanger when a real multicomponent chloride solution is treated.

The observed sorption behaviour can be related to the structure of the exchanger. Lewatit MonoPlus SP112H contains fixed sulfonic groups within a macroporous styrene–divinylbenzene matrix. These groups provide the cation-exchange sites responsible for Sc(III) uptake, while their accessibility and the swelling of the polymer matrix influence the effective resin loading. At the same time, sulfonic groups are not highly selective toward scandium, and competing cations, particularly Fe(III), can interact with the same exchange sites. Therefore, the observed Sc/Fe separation reflects both the chemical nature and accessibility of the functional groups and the composition of the real multicomponent chloride solution. This structure–performance relationship explains the residual iron loading and indicates that resins bearing more selective functional groups may provide improved Sc/Fe separation.

After the first sorption stage the raffinate still contained 96.01 mg Sc, corresponding to about 3.77 mg/L. Treatment of the raffinate with a fresh portion of resin sorbed a further 52.80 mg Sc. This amounted to 55.0% of the scandium remaining after the first stage and to 11.11% of its initial content in the combined filtrate. The scandium capacity of the second resin portion was 0.87 mg/g. The lower capacity compared with the first stage was caused by the lower scandium concentration in solution.

After the two sorption stages, the raffinate contained 43.21 mg Sc at a residual concentration of about 1.80 mg/L. The two resin portions sorbed 432.09 mg Sc in total, corresponding to a recovery of 90.91%. The raffinate also carried Mn at about 1.6 g/L and Cr at about 0.3 g/L, which shows that divalent manganese and trivalent chromium are held only weakly by the resin under the high sodium, potassium and magnesium background and pass into the raffinate. The second stage raised scandium recovery from 79.8 to 90.9%, that is, by 11.1 percentage points.

Desorption from the first resin portion transferred 359.56 mg Sc into solution, a desorption degree of 94.8%. The second resin portion released 50.15 mg Sc, or 94.98% of its resin content. The combined eluates had a total volume of 650 mL and a total scandium content of 409.71 mg. The overall desorption degree of scandium reached 94.82%, and the overall transfer of scandium from the combined filtrate to the eluate was 86.20%. The scandium mass balance along the flowsheet is presented in Table 4.

All scandium losses were calculated relative to the 475.30 mg Sc contained in the combined leach filtrate. The loss during sorption was 43.21 mg (9.09%), corresponding to scandium remaining in the final raffinate. Incomplete desorption resulted in a further loss of 22.38 mg (4.71%) retained on the resin. Oxalate precipitation and calcination accounted for a loss of 19.71 mg (4.15%), leaving 390.00 mg Sc (82.05%) in the intermediate oxide. The subsequent additional purification sequence resulted in a cumulative loss of 95.70 mg (20.13%), leaving 294.30 mg Sc (61.92%) in the final oxide product. The four loss contributions total 181.00 mg Sc (38.08%); together with the final recovery of 61.92%, the scandium balance closes at 100.00%. Because scandium was not quantified separately in each stream of the additional purification sequence, the 95.70 mg value represents the combined loss associated with redissolution, re-sorption, re-desorption, reprecipitation and final calcination. Thus, the 20.13% cumulative loss during additional purification is included in, and is not additional to, the total scandium loss of 38.08%.

Precipitation of scandium as the oxalate followed by calcination gave 0.60 g of an intermediate oxide product. X-ray fluorescence analysis gave the following contents in the product: wt%: 65.0 Sc; 0.10 Fe; 0.10 REE; 0.06 Si; 0.05 Na; 0.03 Ca; 0.02 Mn; 0.02 K; 0.02 S; and 0.01 Al. The calculated scandium content of the intermediate product was 390.00 mg.

The transfer of scandium from the combined eluate to the intermediate oxide product reached 95.19%. The scandium content of the product was close to its theoretical mass fraction in Sc_2_O_3_ of 65.2%, which is consistent with the formation of a scandium oxide-rich product. The purity of the intermediate product should nevertheless be assessed conclusively by full elemental analysis. The oxygen content in this case must be regarded as calculated or obtained by difference rather than as an independently measured value.

**Figure 6:**
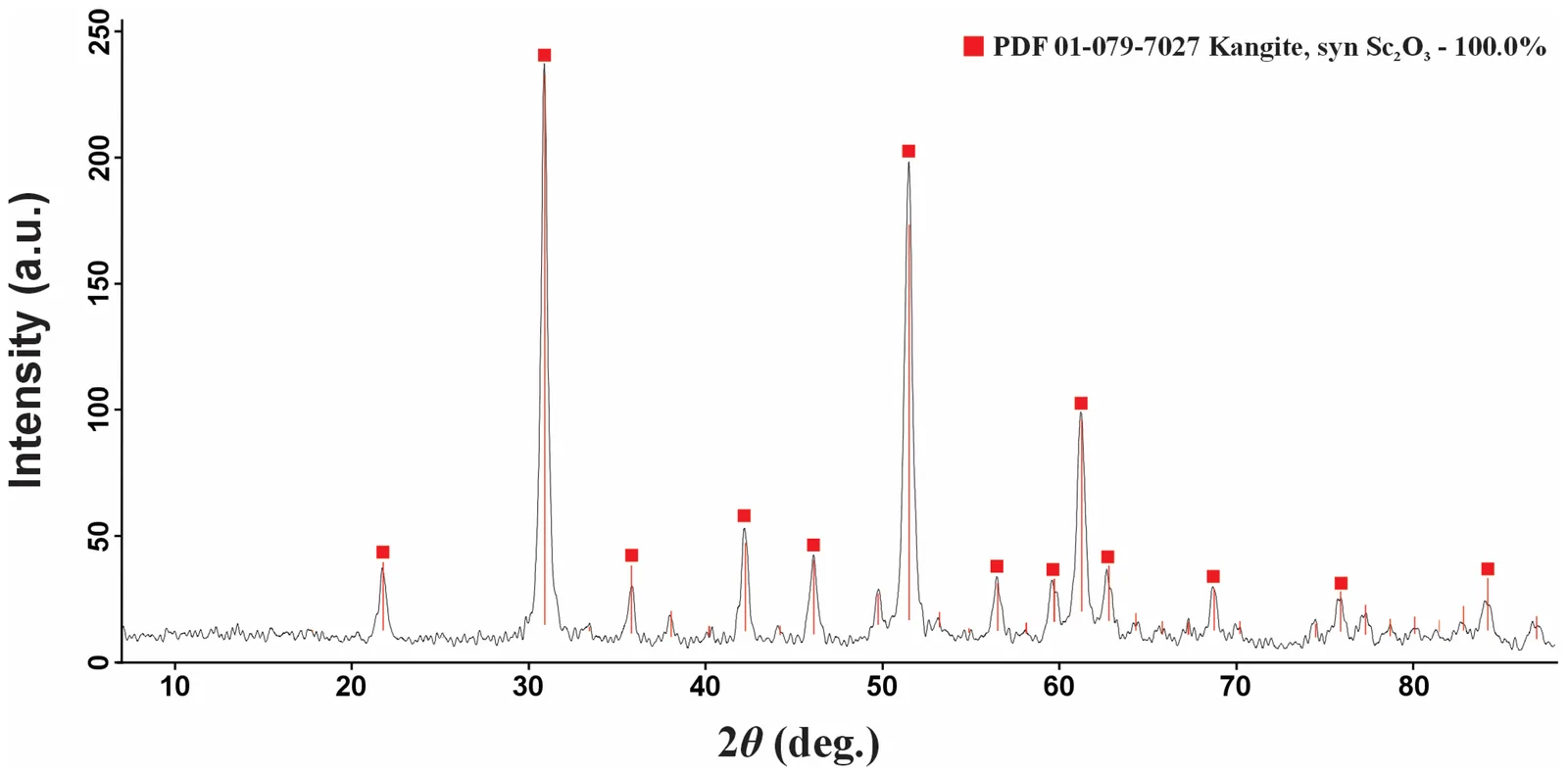
X-ray diffraction pattern of the oxide product.

Additional sorption purification, reprecipitation and calcination gave 0.45 g of the final oxide product. Its diffraction pattern showed reflections corresponding to cubic Sc_2_O_3_ with the bixbyite-type structure. No other crystalline phases were detected within the sensitivity of X-ray phase analysis (Figure 6). X-ray diffraction establishes the predominant crystalline phase and not the chemical purity of the material, since amorphous admixtures and crystalline phases below the detection limit of the method remain outside its reach.

X-ray fluorescence analysis of the final product gave 65.4 wt% Sc, which matches the stoichiometric scandium content of Sc_2_O_3_ (65.2 wt%) and corroborates the phase assignment from XRD. The main impurities were sulfur, 1.0 wt%, and chlorine, 0.96 wt%, carried over from the ammonium sulfate eluent and the hydrochloric redissolution. Titanium, molybdenum and iron were each below 0.05 wt%, and calcium, vanadium, chromium, manganese and the other elements sought lay below detection. The detected impurities total about 2 wt%, from which the purity of the product is estimated at close to 98 wt%. The residual sulfur and chlorine show that more thorough washing and a higher calcination temperature are needed to lower the anion content.

The measured scandium content corresponds to 294.30 mg Sc in the 0.45 g of product, 75.46% of the scandium in the intermediate oxide and 71.83% of the combined eluate. The lost scandium leaves with the column raffinate, the acid wash and the oxalate mother liquor, none of which was recycled in the present tests. Recirculation of the purification raffinate and of the mother liquors is the direct route to lower the losses. The figures for the two oxide products rest on their measured scandium contents and not on an assumed stoichiometric composition.

The two sorption stages left 24.0 L of raffinate containing mainly potassium, sodium and magnesium chlorides together with most of the iron and aluminium not taken up by the resin. After purification with lime milk, the volume of the chloride solution was 25.3 L. The concentrations of the main components in the purified solution were, in g/L: 20.5 KCl; 18.1 NaCl; 13.3 MgCl_2_; 0.005 Fe and 0.001 Al.

**Figure 7:**
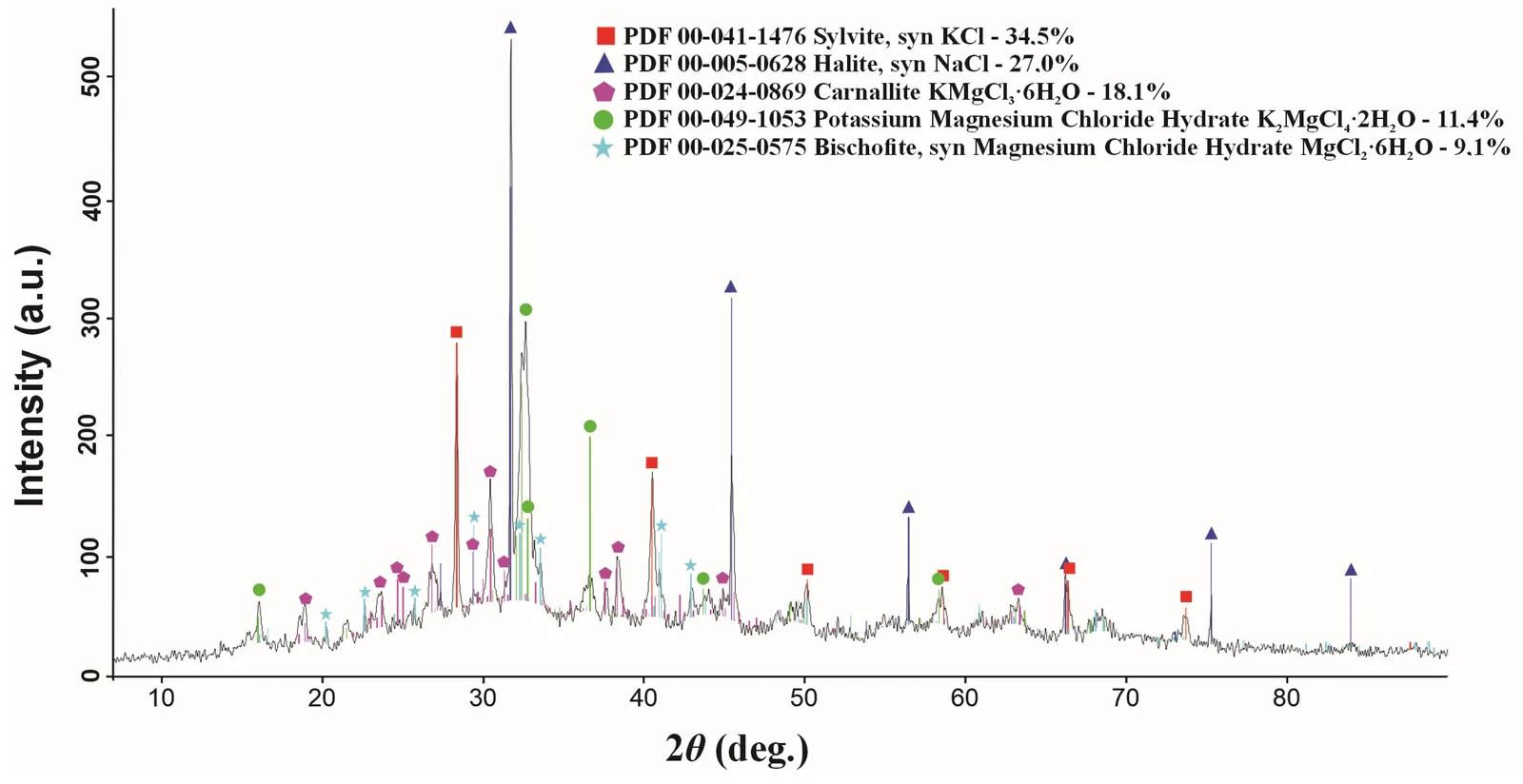
X-ray diffraction pattern of the mixed potassium–sodium–magnesium chloride product.

Adjustment of the KCl/MgCl_2_ ratio and evaporation of the solution gave 1550 g of a mixed chloride salt product. X-ray phase analysis showed it to consist mainly of sylvite, halite and carnallite-bearing phases (Figure 7). The presence of sylvite, halite and several hydrated magnesium-bearing phases indicates that the material obtained was not individual carnallite. It should therefore be described as a mixed potassium–sodium–magnesium chloride product containing carnallite and hydrated magnesium chloride phases.

The scaled-up test thus confirmed the feasibility of successive scandium recovery from STCM with the preparation of intermediate and final oxide products, together with a by-product mixed potassium–sodium–magnesium chloride product obtained from the raffinate.

Table 5 compares the present results with scandium sorbents and extractants reported for chloride and sulfate media. Direct quantitative comparison is limited because the cited studies differ in feed composition, acidity, scandium concentration and impurity levels, and only a few report performance for real multicomponent process liquors. Against this background, the present route is distinguished primarily by its application to a real multicomponent STCM liquor and by the stagewise scandium mass balance. A direct ranking of reagent consumption, energy demand or emissions is not possible from the available literature because these parameters are not reported consistently across the compared studies. Adsorption using engineered composite materials represents an additional approach to metal recovery [31]; however, such systems have been investigated mainly for heavy metals in dilute aqueous solutions rather than for scandium recovery from strongly acidic chloride liquors.

The scaled-up test was performed as a single run using one averaged batch of spent titanium chlorinator melt and one selected strong-acid cation exchanger, Lewatit MonoPlus SP112H. The reported recovery, resin loading and separation characteristics should therefore be considered representative of the investigated material and process conditions and not as a general assessment of strong-acid cation exchangers as a class. Further tests on independent STCM batches and on other sorbents are required to assess the broader applicability of the process. The multi-cycle sorption–regeneration performance of Lewatit MonoPlus SP112H was not evaluated in the present study. Repeated-cycle tests with monitoring of scandium recovery, selectivity and resin capacity are therefore required to assess resin stability and long-term process applicability.

Additional limitations of the present study are that pH and competing-anion effects were not varied independently, multi-cycle resin regeneration was not evaluated, and FT-IR, SEM and EDS characterisation of the resin before and after sorption was not performed. In addition, the Fe(III)/Fe(II) ratio and redox potential were not independently measured during the ascorbic-acid reduction step. These aspects should be addressed in future work to further clarify the sorption mechanism, resin stability and process applicability.

## 4. Conclusions

A sequence for scandium recovery from spent titanium chlorinator melt was developed, comprising hydrochloric acid leaching, Fe(III) reduction, two-stage sorption on Lewatit MonoPlus SP112H, desorption with acidified ammonium sulfate solution, oxalate precipitation, calcination and an additional sorption purification. The highest scandium recovery was obtained at 5% HCl and the highest resin loading at 10% HCl, whereas 3% HCl gave the highest separation factors of scandium over iron and aluminium. The sorption kinetics were described satisfactorily by the non-linear pseudo-second-order model (*R*^2^ = 0.890–0.938). A higher temperature accelerated the accumulation of scandium on the resin, while the shape of the kinetic curves indicated a joint contribution of external and intraparticle mass transfer.

In the scaled-up test, treatment of 4.0 kg of STCM produced 25.5 L of combined filtrate containing 475.30 mg Sc. The first sorption stage recovered 79.8% of the scandium, and re-treatment of the raffinate with a fresh portion of resin raised the total recovery to 90.91%. The overall desorption degree from the two resin portions was 94.82%, and the overall transfer of scandium from the combined filtrate to the eluate reached 86.20%. The Fe:Sc mass ratio decreased from about 386:1 in the initial filtrate to 25:1 in the loaded resin, corresponding to an approximately fifteen-fold enrichment of scandium relative to iron. The high absolute iron loading of the resin, however, indicates the limited selectivity of the process in a real multicomponent chloride solution.

Oxalate precipitation and calcination yielded 0.60 g of an intermediate oxide product containing 65.0 wt% Sc. After additional purification, reprecipitation and calcination, 0.45 g of the final oxide product was obtained with a scandium content of 65.4 wt%. X-ray diffraction confirmed cubic Sc_2_O_3_ as the predominant crystalline phase, while the analytical results indicated a product purity of approximately 98%. The overall scandium recovery into the final oxide was 61.92% relative to the combined filtrate.

A mixed potassium–sodium–magnesium chloride product consisting of sylvite, halite, carnallite and hydrated magnesium chloride phases was also obtained from the raffinate. The results confirm the feasibility of integrated STCM processing with recovery of scandium into an oxide product and simultaneous production of a chloride salt product. Higher sorption selectivity of scandium over iron and lower scandium losses during the additional purification are the main directions for further optimisation. Testing of selectivity-oriented sorbents, such as chelating aminophosphonic resins, on the real leach filtrate, and recirculation of the purification streams, define the next stage of the work.

## Figures and Tables

**Table 1:** Elemental composition of the STCM batch used.

Component	Sc	Fe	Al	Na	Mg	K	Si	Ti	Ca	Cr	Mn	Zr	Cl	O	Moisture
Content, wt%	0.015	9.02	1.01	4.55	2.18	6.87	1.75	1.52	0.81	0.92	1.34	0.13	33.55	13.5	22.46

**Table 2:** Initial Sc concentration, sorption capacity and separation factors on Lewatit SP112H as a function of the HCl concentration.

HCl, %	Initial *C* (Sc), g/L	*q*_Sc_, mg/g	β (Sc/Fe)	β (Sc/Al)
2	0.03300	0.385	8.4	11.4
3	0.04296	0.508	9.6	13.8
5	0.04834	0.870	7.8	7.1
10	0.04788	1.107	4.3	4.0
15	0.03488	1.040	1.9	1.7

**Table 3:** Parameters of the kinetic models of scandium sorption on Lewatit SP112H obtained by non-linear regression.

*T*, °C	Pseudo-First Order			Pseudo-Second Order			
	*q*_e_, mg/g	*k*_1_, min^−1^	*R* ^2^	*q*_e_, mg/g	*k*_2_, g·mg^−1^·min^−1^	*h*, mg·g^−1^·min^−1^	*R* ^2^
20	0.496	0.0258	0.853	0.551	0.0636	0.0193	0.937
40	0.753	0.0366	0.850	0.830	0.0593	0.0408	0.938
60	1.175	0.0296	0.833	1.324	0.0274	0.0480	0.918
80	1.262	0.0414	0.785	1.364	0.0462	0.0858	0.890

**Table 4:** Scandium mass balance along the flowsheet, relative to the combined leach filtrate.

Stream	Sc Mass, mg	Sc Distribution, %
Initial filtrate	475.30	100.00
Sorbed in the first stage	379.29	79.80
Sorbed in the second stage	52.80	11.11
Total sorbed	432.09	90.91
Loss during sorption (remaining in the final raffinate)	43.21	9.09
Desorbed from the first resin portion	359.56	75.65
Desorbed from the second resin portion	50.15	10.55
Recovered in the combined eluate	409.71	86.20
Loss during desorption (retained on the resin)	22.38	4.71
Loss during oxalate precipitation and calcination	19.71	4.15
Intermediate oxide product	390.00	82.05
Cumulative loss during the additional purification sequence	95.70	20.13
Final oxide product	294.30	61.92

**Table 5:** Comparison of scandium sorbents and extractants reported for chloride and sulfate solutions.

Sorbent or Extractant	Solution	Sc Recovery or Purity	Behaviour of Fe	Ref.
Lewatit MonoPlus SP112H (this work)	Real STCM, 5% HCl filtrate	90.9% Sc (two stages)	β(Sc/Fe) up to 9.6; Fe:Sc from 386:1 to 25:1	This work
KU-2-8; Purosorb SAC140H; Purolite C-150H	Model acidic chloride	Lower Sc affinity than SP112H	n/r	[8]
VP OC 1026; TP 260 (P-bearing)	Ti-production chloride	Sc separated from Fe	No appreciable Fe sorption	[23]
Puromet/Purolite MTS9580	H_2_SO_4_ (bauxite residue); U ISL solutions	Selective Sc recovery	Fe left in solution	[25,26]
Ion-exchange resin screening (Toli et al.)	H_2_SO_4_ leach, 12.7 mg/L Sc, 272 mg/L Fe	Up to 89% Sc adsorbed	Fe(III) pre-reduced with iron powder	[19]
Solvent extraction: D2EHPA; Cyanex 923; TBP	Acidic chloride/sulfate	95–96% purity, 6–9 stages	Iron co-extraction limits economics	[11,14,15]
Roasting + 30% HCl + 30% D2EHPA	Ilmenite slag	94% Sc; 99% Sc_2_O_3_	2.2% Fe co-extracted	[17]

## Data Availability

The original contributions presented in this study are included in the article. Further inquiries can be directed to the corresponding author.
